# Maternal Sensitivity Modulates Child’s Parasympathetic Mode and Buffers Sympathetic Activity in a Free Play Situation

**DOI:** 10.3389/fpsyg.2022.868848

**Published:** 2022-04-19

**Authors:** Franziska Köhler-Dauner, Eva Roder, Manuela Gulde, Inka Mayer, Jörg M. Fegert, Ute Ziegenhain, Christiane Waller

**Affiliations:** ^1^Department of Child and Adolescent Psychiatry/Psychotherapy, University of Ulm, Ulm, Germany; ^2^Department of Psychosomatic Medicine and Psychotherapy, University Hospital of Ulm, Ulm, Germany; ^3^Department of Psychosomatic Medicine and Psychotherapy, Nuremberg General Hospital, Paracelsus Medical University, Nuremberg, Germany

**Keywords:** autonomic nervous system, maternal sensitivity, parasympathetic nervous system, sympathetic nervous system, free and structured play

## Abstract

**Background:**

Behavioral and physiological (self-)regulation in early life is crucial for the understanding of childhood development and adjustment. The autonomic nervous system (ANS) is a main player in the regulative system and should therefore be modulated by the quality of interactive behavior of the caregiver. We experimentally investigated the ANS response of 18–36-month-old children in response to the quality of maternal behavior during a mother–child-interacting paradigm.

**Method:**

Eighty mothers and their children came to our laboratory and took part in an experimental paradigm, consisting of three episodes: a resting phase (E1), a structured play phase (E2), and a free play situation (E3) between mothers and their child. Children’s and mother’s heart rate (HR), the sympathetic nervous system (SNS) activity *via* the pre-ejection period (PEP) and the left ventricular ejection time (LVET), and the parasympathetic nervous system (PNS) activity *via* the respiratory sinus arrhythmia (RSA) were continuously measured by an electrocardiogram. Maternal sensitivity of interactive behavior was assessed by using the Emotional Availability (EA) Scales.

**Results:**

Children of mothers with insensitive behavior had a significantly lower RSA at baseline, showed a lack of RSA withdrawal during structured and free play, and had shorter LVET across all episodes compared to children of sensitive mothers.

**Conclusion:**

Our findings depict the influence of low-quality maternal interaction on the child’s ANS regulation, in calm and more stressful play situations. The overall higher SNS mode with impaired PNS reactivity may negatively influence child’s ANS homoeostasis, which may result in a long-term impact on mental and physical wellbeing. Further, the maternal sensitivity may function as a buffer for the stress response of their child. These results could serve as a basis for the development of appropriate psychoeducational programs for mothers of low sensitivity in their interaction with the child.

## Introduction

The first years of a child’s life represent an essential time for the development of behavioral and physiological regulatory processes associated with healthy socio-emotional and cognitive development (Vohs and Baumeister, 2010). One of the most important points of child development represents the establishment of (self-)regulatory abilities (Posner and Rothbart, 2000; Charles and Carstensen, 2007; Perry et al., 2014). Although infants already have rudimentary self-regulatory abilities, parents play a critical role in shaping children’s emotional, behavioral, and physiological regulatory patterns, especially in the first years of a life (Spangler and Grossmann, 1993; Spangler et al., 1994; Gunnar and Donzella, 2002). Numerous studies suggest that children acquire and develop lifelong psychological and behavioral regulation strategies in the context of early attachment experiences (Bowlby, 1969; Johnson et al., 2007; Smith et al., 2016).

Various theoretical perspectives suggest that sensitive and responsive caregiving, defined by an accurate interpretation and prompt response to infant needs, can facilitate the organization of the response systems of infants and children in favor of a regulation that supports processes of more complex social, emotional, and cognitive development (Schore, 2000; Panksepp, 2001; Siegel, 2001).

The external regulation of children’s regulatory processes becomes particularly relevant in stressful situations if children have not yet developed a sufficient repertoire of regulatory capacities. There seems to be overwhelming agreement that children who experience a predominantly sensitive and responsive caregiving demonstrate healthier patterns of physiological and behavioral strategies both at rest and when facing a challenge or stressor (Calkins and Leerkes, 2011; Propper and Holochwost, 2013; Tabachnick et al., 2019).

Only few research has focused on the quantification of physiological parameters during mother–child interaction (Spangler and Grossmann, 1993; Spangler et al., 1994; Enlow et al., 2014; Köhler-Dauner et al., 2019; Roder et al., 2020). In this context, the autonomic nervous system (ANS) can be considered as one of the most relevant biophysiological measures in this context (Waters and Mendes, 2016; Köhler-Dauner et al., 2019).

The ANS, controlled by the central autonomic network, is mainly involved in emotional and behavioral reactions initiated by an attachment stressor. Therefore, it is particularly relevant to investigate emotion regulation during infant development and in different psychopathologies (Cassidy and Shaver, 1999; Cacioppo et al., 2008; Hill-Soderlund et al., 2008).

The ANS supports maintaining homeostasis and facilitates physiological responses to environmental demands (Tabachnick et al., 2019). Stress-related changes in the ANS are recognizable and measurable long before the awareness of certain emotions, whereby early, unconscious body-related regulation processes can be identified *via* the ANS (Garfinkel et al., 2016; Roder et al., 2020). While ANS activity is thought to describe the general state of the body and its overall capacity for reactivity (El-Sheikh et al., 2010), an individual’s physiological response to a given environmental condition can be assessed by ANS reactivity (Köhler-Dauner et al., 2019; Roder et al., 2020).

The ANS contains two branches—the sympathetic nervous system (SNS) and the parasympathetic nervous system (PNS)—which consist of two different anatomical structures with distinct pathophysiology (Fox and Card, 1999; Neuhuber, 2009). Thus, at the anatomical level, the ANS includes the sympathetic border cord, the nerve plexuses of the chest, abdomen, and pelvis, and the area of the cerebral ganglia as well as parts of the cranial nerves (Neuhuber, 2009). In contrast to the SNS which is responsible for adapting to stressors through the mobilization of the body and behaviors such as the “fight or flight” reaction to an external threat, the PNS is responsible for and mediates the regeneration of the organism and the build-up of energy reserves according to “rest and digest” (Turner, 1989; Larsen et al., 2008; Jänig, 2010; Hoth and Wischmeyer, 2012). SNS activity can be measured by the electrocardiogram (ECG) parameters pre-ejection period (PEP) and left ventricular ejection time (LVET), whereas the PNS activity can be measured by the respiratory sinus arrhythmia (RSA). Activities of the SNS as well as the PNS influences the sinus node of the heart which controls the heart rate (HR). In contrast to adolescents and adults, children vary in their ability to autonomously regulate stress depending on age, environment, emotion regulation, and attachment quality (Hill-Soderlund et al., 2008; Alkon et al., 2011; Michels et al., 2013; Smith et al., 2016), which is why a consideration of the listed parameters especially in childhood is of particular interest.

Haley and Stansbury (2003) found that children of more responsive parents showed more evidence of regulation compared to children of less responsive parents, specifically more regulation of HR. They identified parent responsiveness as a moderator in the change of HR between the still face (stressor episode) and reunion episodes (stress less episode): the HR of children whose parents were in the high responsiveness group decreased whereas it increased for the group of children whose parents were categorized as less responsive (Haley and Stansbury, 2003). Subsequently, Conradt and Ablow (2010) observed more regulation of children’s HR with increasing maternal sensitivity during the reunion episode (stress less episode) of the SFP. In contrast, children of less sensitive mothers had a significantly higher mean HR (Conradt and Ablow, 2010), indicating a higher level of stress and alertness.

Further, RSA has been widely measured in early childhood differing from calm vs. challenging situations (Calkins et al., 2008; Alkon et al., 2011). RSA is purported to index how the heart is influenced by the PNS and represents a psychophysiological indicator for the regulation of arousal, state, and reactivity (Stifter et al., 1989; Porges, 1991; Smith et al., 2016). An elevated RSA at rest is associated with maintaining an adaptive physiological state under sufficient parasympathetic influence and therefore an increased PNS activity (Porges et al., 1994; Porges, 1995; Hastings et al., 2014). In contrast, low baseline RSA has been connected to psychopathology and less flexible responses to social and cognitive challenges. Alkon et al. (2011) found an increase in RSA and thus increased vagal activity with increasing age of the children studied, who were between 6 and 60 months of age. Furthermore, increased vagal regulation was found in children when they were asked to perform tasks together with their mother instead of alone. This regulation was particularly difficult for children with a poorer quality of relationship with their mothers (Calkins et al., 2008). The vagal regulation of children of more insensitive mothers was also found to be less adaptive in a study by Moore et al. (2009). Thus, these children retracted vagally more strongly during free play episodes and weaker during stressful situations. Moreover, they found it more difficult to return to normal levels of vagal activation after such a stressful situation. The development of this vagal regulation is influenced by the emotional support of the mother, among other factors (Perry et al., 2014). If mothers were more responsive and sensitive in their interactions with their infants, they exhibited increased vagal withdrawal than infants of mothers who interacted less responsively and sensitively.

Only few studies focused on the quantification of PEP and LVET, especially in young children. Roder et al. (2020) studied RSA, PEP, and LVET and identified distinct SNS and PNS patterns in one-year-old children during strange situation test (Köhler-Dauner et al., 2019; Roder et al., 2020). LVET, a frequency-related parameter, has been identified as a suitable SNS activity marker especially in early childhood, as younger children can maintain their autonomic balance *via* a sharp change in heart rate (Roder et al., 2020). Since LVET describes a certain period of time, in this case, an increased LVET value means a decreased activity of the SNS, which should be kept in mind. Further, regarding SNS activity, Johnson et al. (2017) observed that the sympathetic branch of the ANS, indexed by PEP, appeared to be more sensitive to a background of adversities in addition to stress than vagal tone whereas maternal responsivity affected the trajectory of PEP activity favorably. Also, in the case of PEP, a higher value describes a reduced sympathetic activation and thus a lower level of alertness.

However, the activity of the ANS can be considered not only with the parameters PEP, LVET, RSA, and HR just described and used for this study, there are also other possibilities such as heart rate variability, respiratory rate, intrinsic heart rate, or electrodermal responses like skin conduction rate (Lazarus et al., 1963; Shields, 1983; Pomeranz et al., 1985; Marcus et al., 1990; Kim et al., 2018).

In the context of this study, we restricted ourselves to the four parameters PEP, LVET, RSA, and HR, because these represent a balanced mixture of already widely researched and less well-documented parameters for ANS activity in children at a very young age. In addition, different methods were used to record the maternal interaction quality in past studies. This contradiction makes it much more difficult to compare and interpret the data together. Beyond that, ANS reactivity rather than resting ANS has been the focal point (Johnson et al., 2017). The aim of our study is therefore to investigate mother–child interaction quality during simultaneous ANS measures with a distinct quantification of PNS and SNS in an emotionally positive approach by using a structured interaction paradigm. Especially the change in PNS and SNS activities between the episodes is of special interest, as this has not been studied so far (Enlow et al., 2014). By that, the influence of the maternal interaction quality on the autonomic stress reactivity of the children can be understood better for future studies. We expect children of more sensitive mothers to regulate their stress better than children of insensitive mothers during structured and free play episodes, indicating a more balanced parasympathetic stress response with higher RSA stability. Furthermore, we assume the SNS activation measured by the LVET to be significantly increased in children of less sensitive mothers. In particular, these assumptions are based on the findings of previous studies that more appropriate responses to stress in children occur when caregiving is more responsive (Calkins and Leerkes, 2011; Propper and Holochwost, 2013; Tabachnick et al., 2019). However, because few studies have been conducted to investigate instantaneous physiological responses to a wide variety of interactional situations in young children, this study aims to help fill this gap.

## Materials and Methods

### Study Design and Participants

TransGen is an interdisciplinary study consortium examining protection and risk factors regarding a transgenerational transmission of childhood maltreatment (CM) by focusing on psychological, biological, and social factors in a prospective design. The study was funded by the Federal Ministry of Education and Research (BMBF, 2013–2016, additional interim funding 2017) and approved by the Ethics Committee of Ulm University, performed in accordance with relevant guidelines and regulations. The main aim was to identify mechanisms of stress resilience and processes in the transgenerational transmission of CM on different levels: psychological, biological, and social. Because this is a longitudinal study, data from this study have been analyzed with respect to ANS activity in previous articles: These are Roder et al. (2020) and Köhler-Dauner et al. (2019).

From October 2013 to December 2015 1,460 healthy women in the maternity unit of the women’s hospital of the University Hospital of Ulm were asked for study participation 1–6 days after parturition of a healthy child. Based on exclusion criteria (age under 18 years, under 37 weeks of pregnancy, insufficient knowledge of the German language, severe complications during parturition, health problems of mother and/or infant, current drug consumption, or history of psychotic disorders or current infections), 533 mothers signed an agreement for participation.

All participating mother–child dyads were then invited for three follow-up time points (t1, t2, and t3). 240 mothers participated in a laboratory and home visit (t1) 3 months post-partum, 158 mother–child dyads took part at a laboratory and home visit around 12 months (t2), and 152 between 18 to 36 months (t3) of child’s age. t3 was the measurement point, where the data for this paper origin from. Sample attrition from t1 to t3 was attributed to personal reasons, lack of interest, and time conflicts for carrying out observations.

Mothers’ age at measurement point t3 was between 27 and 46 years [*M* = 36.6 years (SD = 4.1 years)] and child’s age varied between 1.6 years and 3.7 years [*M* = 2.9 years (SD = 0.1 years)]. The level of education showed that 72.5% of the mothers had completed a grammar school degree (13 years of school), 22.5% a secondary school degree (10 years of school), and 5.0% a basic secondary school degree (9 years of school). Furthermore, 63.7% had achieved at least a bachelor’s degree at a university or a university of applied sciences.

Complete data of ANS measurements during the laboratory paradigm could be collected for 80 children (46 boys and 34 girls) and 72 mothers. Missing or incomplete data sets of mother’s derived autonomous stress reactivity resulted from non-divorcing spot electrodes or the distortion of measured values due to motion artefacts.

### Procedures and Measures

#### Laboratory Paradigm

The study took place in the rooms of the research department of the Clinic for Child and Adolescent Psychiatry/Psychotherapy at the University Hospital Ulm. For the implementation of the paradigm, an experimental empathy paradigm was developed, based on the paradigm processes of Doris Bischof-Köhler (1994, 1998, 2012). The paradigm was divided into eight episodes of different lengths (from 30 s to 10 min). For the findings presented here, it should be noted that only the first three episodes (E1–E3) of the empathy paradigm were part of the investigation, as only the three episodes described below are relevant for considering the effects of stress induction and stress reduction.

As has already been established in previous studies (Tronick et al., 1978; Calkins et al., 2008; Alkon et al., 2011), the study of parental sensitivity can best take place in conflictual play episodes. Such conflictual episodes are characterized by concrete challenges, that need to be worked on together. In this case, the challenge was solving a given learning puzzle together with the mother. Often these play paradigms are divided into three episodes: Resting period (E1), Structured Play (E2), and Free Play (E3). This procedure was implemented in this study as follows: Mothers and children were invited between 9 AM and 11 AM or in the afternoon between 1 PM and 5 PM After a short introductory talk, seven spot electrodes for measuring ECG and impedance cardiogram (ICG) were applied to the mother and child as published elsewhere (Roder et al., 2020) and recording started. The first episode was a 5-min resting period for baseline measurement (E1). Mothers took a seat in the anteroom of the examination room and were asked to relax while the children played with a toy of their choice. E1 was designed based on the baseline episodes of similar paradigms (Skowron et al., 2011; Bush et al., 2016; Smith et al., 2016; Roder et al., 2020).

**Figure 1:**
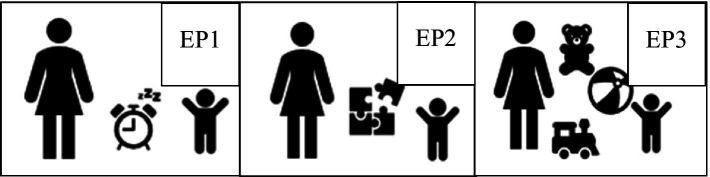
Illustration of the episodes of the laboratory paradigm. EP1—resting phase: Relaxing of mother and child in the anteroom while child plays with toy of choice to measure baseline ANS activity. EP2—structured play: Solving a learning puzzle together (stress inducing due to specific task). EP3—free play: Playing together with toys of choice (stress relieving due to no specific task).

E2, consisting of a structured play phase, contained two age-appropriate learning puzzles. Mothers were asked to play with them together with their child for the next 10 min. This procedure was applied to induce stress due to the specific task of solving a puzzle. Then, children were allowed to choose a toy of their choice from the examination room and play with it for the next 10 min in E3 (free play phase). In this part of the paradigm, the previously built-up stress, induced by the challenge of solving the puzzle, should be relieved. Finally, the experimenter entered the room and, together with mother and child, tidied up the previously used toys in order to prepare for the subsequent episode, which is not part of this paper. Figure 1 illustrates the here used three episodes of the laboratory paradigm.

#### Quality of Parental Behavior

The Emotional Availability (EA) scales (Biringen and Easterbrooks, 2012) were used to assess the quality of the parental behavior during the three described play episodes of t3. These measure the quality of parent–child interactions with consideration of the adult’s “receptive presence” to the child’s emotional signals (Biringen and Easterbrooks, 2012). The scales assess the quality of a dyadic relationship, for example, between mother and child, whether they succeed in developing an authentic and emotionally positive bond, as well as being able to balance and regulate negative emotions. They consist of a total of six subscales. Four of the subscales describe the caregiver’s style of interaction with the child: Parental Sensitivity, Parental Structuring, Parental Nonintrusiveness, and Parental Nonhostility. Two of the subscales record the child’s behavior toward the caregiver: Child Responsiveness and Child Involvement. Multimodal coding refers to verbal and non-verbal aspects of an interaction such as facial expression, tone of voice, body language, and affects (Biringen and Easterbrooks, 2012).

The EA Scales are coded using 5–9-point scales. The focus of the analyses presented here related to the subscale “Parental Sensitivity,” which measures the parental ability to understand child signals, to respond appropriately to child signals, affect, timing, variety, and creativity in play and flexibility in play conflict resolution.

The point values for the “Parental Sensitivity” measure from 1 (“highly insensitive”) to 9 (“highly sensitive”). The scaling also enables a dimensional division into “sensitive” (scale values from 5.5 to 9.0) and “less sensitive” (scale values from 1.0 to 5.0) parental behavior (Biringen et al., 2000). Three coders scored all play sessions and were blind to all other data of the mother–child dyads. The assessments of all three coders were used to calculate a mean value for each mother. The interrater reliability, calculated with Kendall’s concordance coefficient and then transformed in Spearman’s correlation, was sufficiently large according to the recommendation of Cohen (1988) with a correlation higher than *ρ* = 0.50. The coders were trained by and reliable with the original developers of the EA scales (Biringen and Easterbrooks, 2012).

#### ANS Measures

During the entire paradigm, ECG and ICG were recorded simultaneously and continuously in mothers and their children by using wireless lightweight mobile units (Mindware Technologies, Gahanna, United States) during the whole paradigm described above. The measurement method using spot electrodes and mobile units in a simultaneous manner and especially for one-year-old children has been described elsewhere (Roder et al., 2020).

HR, PNS activity *via* the RSA, and SNS activity *via* the PEP and LVET were determined as follows: HR was derived from the interbeat intervals using the ECG (Alkon et al., 2003). RSA was determined as an index from the interbeat intervals of the ECG and the respiratory rates derived from the ICG (Bar-Haim et al., 2000). PEP refers to the time interval in ms from the beginning of the ventricular depolarization until the opening of the aortic valve (Alkon et al., 2003). LVET resulted from the time interval during systole until the closure of the aortic valve, derived from the ICG (Thayer and Uijtdehaage, 2001).

#### ANS Analysis

An average of ten 30-s segments were determined for E1. Twenty 30-s segments were determined in all episodes of the paradigm, respectively. An average of all 30-s segments were calculated for each episode and value for further statistical analyses.

The ECG and ICG data collected were cleaned up in two steps using the Heart Rate Variability Analysis 3.1.1 as well as the Impedance Analysis 3.1.1 (MindWare Technologies) programs and were carried out by trained employees. All data cleaning procedures, including surveillance at random, were implemented, as already in Roder et al. (2020) paper described for an earlier measurement date.

### Statistical Analyses

Data were analyzed using Statistical Package for the Social Sciences version 27.0 (SPSS Inc., Chicago, IL). Statistical significance was set at *p* < 0.05 (two-tailed). Descriptive statistics were calculated to examine the variables’ distributions and characteristics.

Normal probability plots and the Kolmogorov–Smirnov test showed that our data were normally distributed except for the LVET data and child age. However, the performed analyses can be considered robust against violations of the normal distribution assumption (Schmider et al., 2010; Blanca et al., 2017).

We reviewed the bivariate association between control and key study variables ahead of our main analyses by calculating Pearson correlations. Inferential analyses were conducted as follows: an analysis of covariance (ANCOVA) for repeated measures was performed for each ANS parameter (HR, RSA, LVET, and PEP) for child as well as for mother between subjects (group: “not-sensitive”/“sensitive” maternal interaction behavior) and within subjects (E1–E3) with child sex and child age as covariates. Greenhouse–Geisser correction for repeated measures was applied when the Mauchly W-test indicated a significant deviation from sphericity.

## Results

### Descriptive Analyses

Complete data of ANS measurements could be collected for *N* = 80 children (46 boys and 34 girls) and *N* = 72 mothers. 52 children (32 boys and 20 girls) were classified to have sensitive whereas 28 (14 boys and 14 girls) were classified to have insensitive mothers. The descriptive data as well as χ^2^ tests are presented in Table 1.

**Table 1:** Descriptive data and results of *χ*^2^ tests.

		***N***	**%**		***χ*** ^**2**^	**df**	***p***
**Child sex male**		46	57.5	*χ* ^2^ test	0.99	1	0.319
**Maternal sensitivity**		52	65.0				
				*χ* ^2^ test	0.64	1	0.425
**Child age young (age < 2.8 years)**		42	52.5				
		**M**	**SEM**	**SD**	**Median**	**Min**	**Max**
**Child age**		2.9	0.1	0.1	2.8	1.6	3.7
**Mother age**		36.6	0.1	4.1	36.4	27.0	45.9
**HR**	E1	116.5	1.2	10.7	115.1	72.8	142.4
	E2	118.7	1.1	9.7	118.5	73.7	142.1
	E3	119.6	1.2	10.9	119.0	74.1	147.6
**RSA**	E1	5.3	0.1	0.9	5.2	3.0	7.4
	E2	5.1	0.1	0.7	5.0	3.7	7.2
	E3	5.0	0.1	0.8	5.0	3.0	7.1
**LVET**	E1	204.4	3.9	34.8	216.3	95.7	253.3
	E2	205.9	3.5	31.1	214.0	138.0	264.0
	E3	203.2	3.5	30.9	210.7	94.3	248.8

*χ*^2^ tests are, respectively, comparing children of insensitive vs. sensitive mothers regarding gender and age. Other statistical values are referring to whole (child) sample each. Abbreviations: M, mean value; SEM, standard error of the mean; SD, standard deviation; Min, minimum; Max, maximum; HR, heart rate; RSA, respiratory sinus arrhythmia; LVET, left ventricular ejection time; E1, resting phase; E2, structured play; E3, free play.

### Correlation Analyses

HR was negatively related to child’s age across all three episodes [E1: *r*(78) = −0.49, *p* < 0.001, E2: *r*(78) = −0.49, *p* < 0.001, and E3: *r*(78) = −0.48, *p* < 0.001] and RSA showed a positive association with child age in E1 [*r*(78) = 0.23, *p* < 0.05] and E3 [*r*(78) = 0.36, *p* < 0.001].

RSA was found to be positively correlated with maternal sensitivity in E1 [*r*(78) = 0.41, *p* < 0.001] and E2 [*r*(78) = 0.43, *p* < 0.001], and showed an inverse correlation in E3 [*r*(78) = −0.47, *p* < 0.001]. LVET showed a positive correlation with maternal sensitivity in E1 [*r*(78) = 0.48, *p* < 0.001], E2 [*r*(78) = 0.56, *p* < 0.001], and E3 [*r*(78) = 0.48, *p* < 0.001]. Mother’s age correlated negatively with their HR in E2 [*r*(73) = −0.25, *p* = 0.034] and E3[*r*(72) = −0.29, *p* = 0.016].

### Child’s HR and Maternal Sensitivity

When comparing children’s HR across all episodes between sensitive and insensitive mothers, the main time, group and group x time interaction effects were not significant [*F*(1.74, 132.37) = 2.03, *p* = 0.141, $\eta_{p}^{2}$ = 0.026, *F*(1, 76) = 0.00, *p* = 0.951, $\eta_{p}^{2}$ = 0.00 and *F*(1.74, 132.37) = 0.12, *p* = 0.863, $\eta_{p}^{2}$ = 0.00, respectively].

### Child’s RSA and Maternal Sensitivity

**Figure 2:**
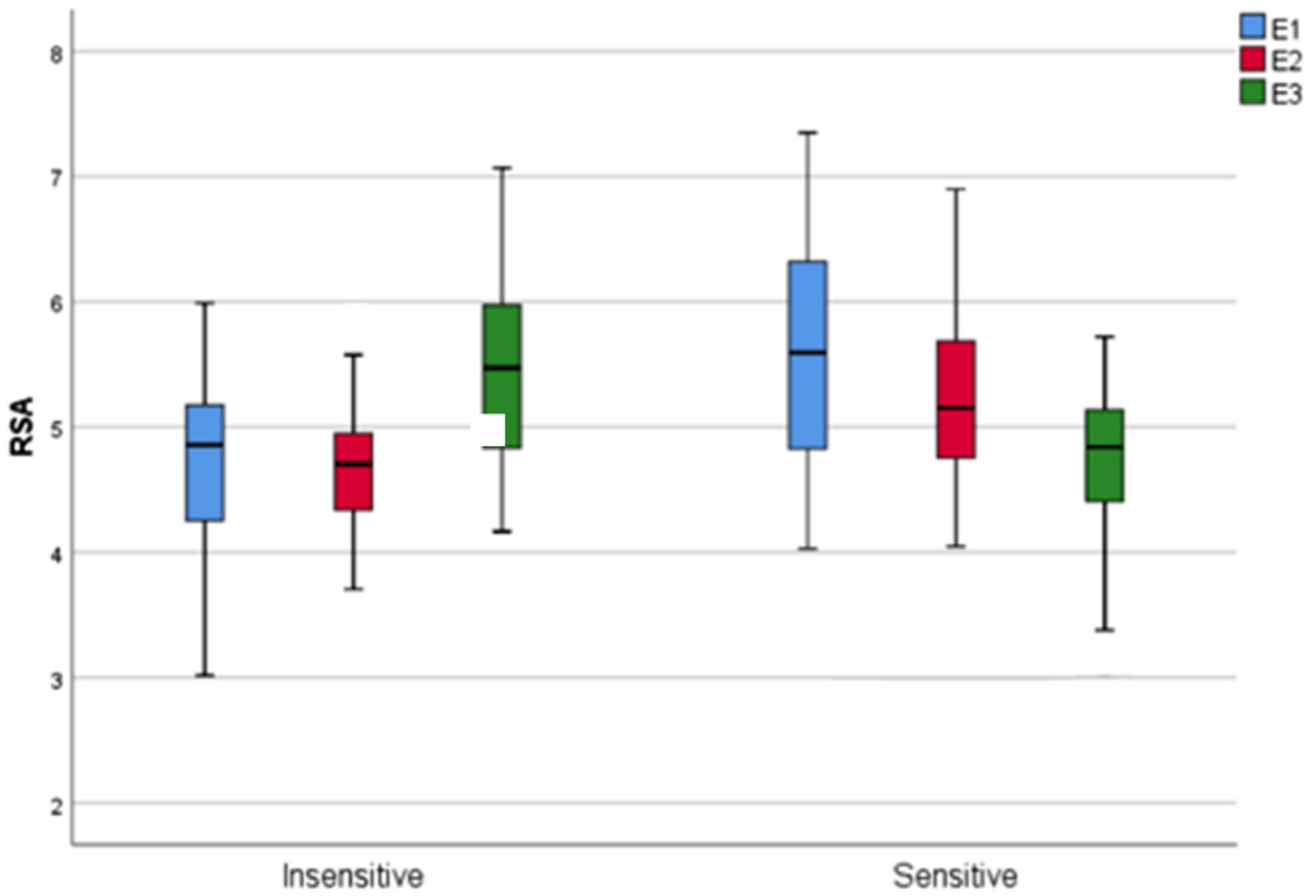
Differences in children’s RSA depending on maternal sensitivity across episodes. RSA is measured as an index calculated by using interbeat intervals and respiratory rates. RSA, respiratory sinus arrhythmia.

Whereas the main time effect of children’s RSA across all episodes between sensitive and insensitive mothers was not significant [*F*(2, 152) = 0.70, p ‚ = 0.498, $\eta_{p}^{2}$ = 0.009], the main group effect showed significant differences [*F*(1, 76) = 5.10, *p* = 0.027, $\eta_{p}^{2}$ = 0.06] with higher RSA values in children with sensitive mothers. The interaction of group x time was also significant [*F*(2, 152) = 34.09, *p* < 0.001, $\eta_{p}^{2}$ = 0.31]. Thus, the RSA value in children of insensitive mothers was lower than in children of sensitive mothers in the first episode, but in the third episode, there was an increase in RSA in children of insensitive mothers and a decrease in children of sensitive mothers. The interaction effect is illustrated in Figure 2.

### Child’s LVET and Maternal Sensitivity

**Figure 3:**
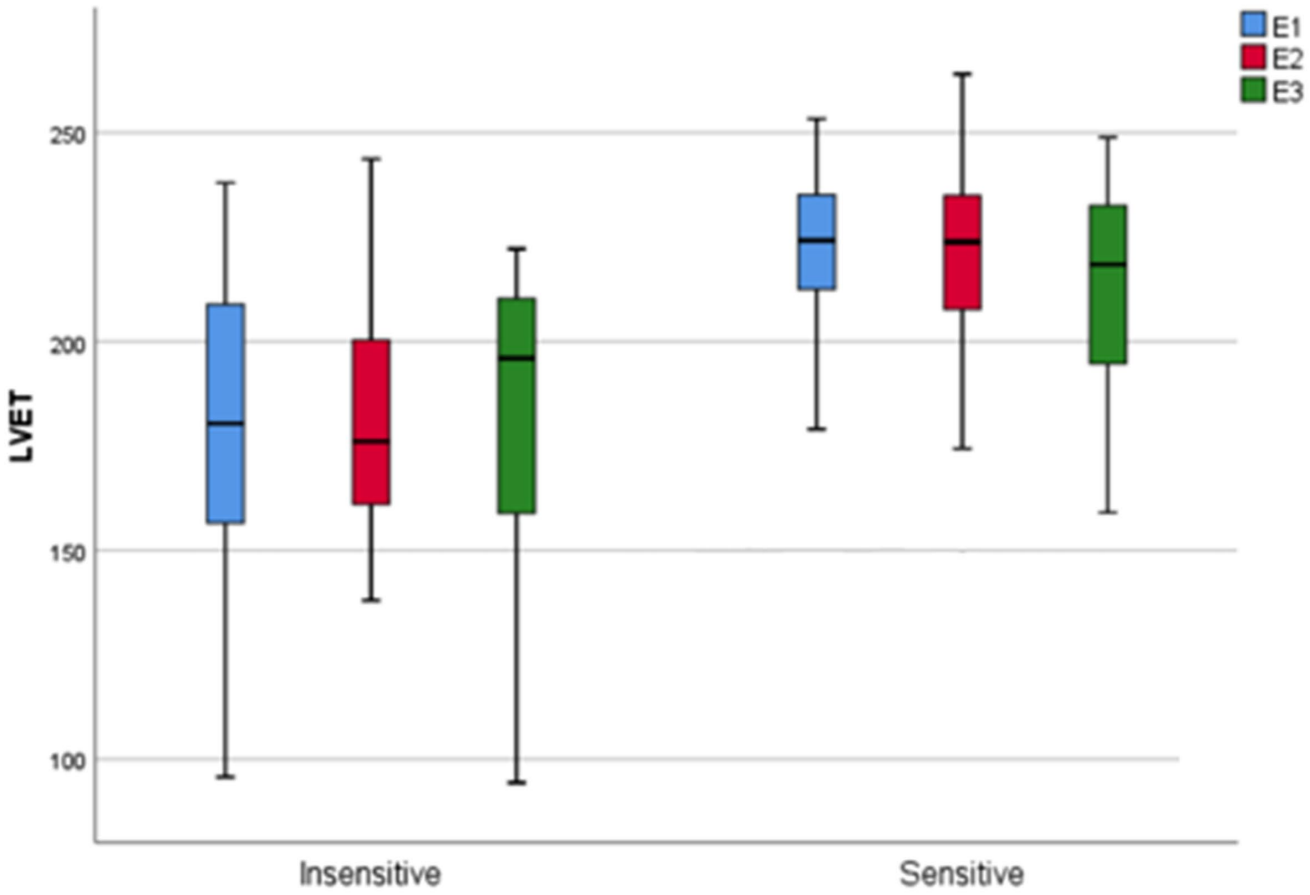
Differences in children’s LVET depending on maternal sensitivity across episodes. LVET is measured in ms. LVET, left ventricular ejection time.

The main time effect was not significant [*F*(2, 152) = 0.70, *p* = 0.489, $\eta_{p}^{2}$ = 0.009] when comparing children’s LVET values across the episodes. However, the main group effect showed significance [*F*(1, 76) = 36.43, *p* < 0.001, $\eta_{p}^{2}$ = 0.324] with longer LVET values in children with sensitive mothers. The group x time interaction effect was not significant [*F*(2, 152) = 0.29, *p* = 0.747, $\eta_{p}^{2}$ = 0.00]. The results are also illustrated in Figure 3.

### Child’s PEP and Maternal Sensitivity

When comparing children’s PEP values, there was no significant effect over time [*F*(2,152) = 0.40, *p* = 0.669, $\eta_{p}^{2}$ = 0.01], group comparison [*F*(1, 76) = 0.05, *p* = 0.839, $\eta_{p}^{2}$ = 0.00], or interaction [*F*(2,152) = 1.06, *p* = 0.350, $\eta_{p}^{2}$ = 0.01].

### Mothers’ HR, RSA, LVET, PEP, and Maternal Sensitivity

No significant results were detectable in the ANCOVA comparing mother’s HR, RSA, LVET, or PEP across the episodes between sensitive and insensitive mothers. The results are shown in Table 2.

**Table 2:** Results of the ANOVA of mother’s HR, RSA, LVET, and PEP.

	df1	df2	*F*	*p*	$\eta_{p}^{2}$
HR					
Time effect	1.64	112.07	0.7	0.474	0.01
Group effect	1	68	0.22	0.644	0
Interaction effect	1.64	112.07	0.43	0.613	0.01
RSA					
Time effect	2	134	0.22	0.805	0
Group effect	1	67	0.74	0.393	0.01
Interaction effect	2	134	1.01	0.366	0.02
LVET					
Time effect	2	136	0.35	0.705	0
Group effect	1	68	0.01	0.934	0
Interaction effect	2	136	0.39	0.681	0.01
PEP					
Time effect	2	136	0.43	0.649	0.01
Group effect	1	68	0.42	0.519	0.01
Interaction effect	2	136	0.62	0.542	0.01

HR, heart rate; RSA, respiratory sinus arrhythmia; LVET, left ventricular ejection time; PEP, pre-ejection period.

## Discussion

Our results show that children of insensitive mothers had lower overall RSA scores, indicating lower PNS activity and difficulties in maintaining the homeostatic state. This result is consistent with current literature (Moore, 2010; Enlow et al., 2014; Johnson et al., 2017; Köhler-Dauner et al., 2019), which indicate that higher quality of maternal caregiving helps children to regulate their homeostasis and therefore regulate themselves effectively. In particular, children with low RSA levels are later particularly affected by the effects of sensitive parenting: for example, a positive association between sensitive parenting in various play tasks and executive functions in preschool was found only among children who had low RSA scores (Gueron-Sela et al., 2017). Thus, these children in particular appear to be dependent on external regulation to develop appropriately. The fact that in this sample the children of insensitive mothers had lower RSA scores on average should therefore be considered highly problematic.

Second, children of sensitive mothers showed significantly higher RSA decrease during structured and free play than children of insensitive mothers. This overall decrease in RSA indicates that in this sample children of sensitive mothers regulate themselves particularly *via* the PNS, whereas children of insensitive mothers more *via* the SNS. Since the PNS is responsible for a person’s regeneration and recovery, and the SNS is responsible for maintaining constant alterness and preparation for potential threats (Turner, 1989; Larsen et al., 2008; Jänig, 2010; Hoth and Wischmeyer, 2012), it becomes clear that children of insensitive mothers exhibit an unhealthy form of adaptation to the stressors in the paradigm through SNS activation.

Further, RSA withdrawal is thought to reflect adaptive PNS regulation in order to increase arousal needed for tasks engagement (El-Sheikh et al., 2009). Therefore, the significant RSA withdrawal as reaction to the cognitive and interactional challenge during structured play can be attributed to better regulatory abilities of children of sensitive mothers. This finding corresponds with Enlow et al. (2014) that children of insensitive mothers exhibited lower PNS activation during stress. This further demonstrates that structured play is capable of inducing stress in children. Interestingly, RSA of children with insensitive mothers showed a further increase from structured to free play situation with the mother compared to children of sensitive mothers (Figure 2). This may mean that children of insensitive mothers show an increase in homeostasis in the sense of calming down with the start of free play, although the free space now given in the design of play should actually excite the child positively (Kwon et al., 2013). For example, Kwon et al. (2013) found in their study that normally children engaged in more complex and focused play behaviors in free play with their parents than in structured play. Parents also exhibited significantly less negative parenting behavior in free play, which may explain the child’s increased willingness to be more active in free play than in structured play (Kwon et al., 2013). However, according to our physiological measurement, children of insensitive mothers did not show this expected behavior in free play, which may be explained by a poorer quality of parenting behavior of such mothers in free play. This can also be seen as sign of relief to be able to move freely in the room and to withdraw from the direct interaction with the less sensitive mother. As a result of this relief of being able to distance themselves from the insensitive mother, there was a calming in the child in the form of even more increased PNS activity. As mothers of higher sensitivity are better at responding to children’s signals, their children might not experience as much stress during the structured play. This suggestion corresponds with Enlow et al. (2014) report that infants of predominantly sensitive mothers showed lower levels of distress across episodes of strange situation test even though infants’ overall distress increases during the still face and decreased during the reunion episodes. Therefore, this pattern may implicate that maternal sensitivity functions as a buffer to stress reactivity (Enlow et al., 2014). This finding is also consistent with the findings of our group (Köhler-Dauner et al., 2019; Roder et al., 2020). In that study, we found an increase in the RSA of children with disruptive mothers in a situation in which such a reaction was rather unexpected (Köhler-Dauner et al., 2019).

Third, children of mothers with less sensitive behavior had significantly shorter LVET across all episodes, indicating higher SNS activity. It appears that a lower quality of interaction behavior from the mother over the first years of a child’s life contributes to a chronically lower level of SNS activation. If this were the case, this would be a risk for the individual variability of the ANS, and therefore adaptability to stress (Chambers et al., 2015). This is also consistent with the finding of Enlow et al. (2014): in their study, children of insensitive mothers experienced significantly greater distress over the course of the Still Face Paradigm and higher SNS activation. Roder et al. (2020) found that the derivation of LVET for the description of children’s stress reactivity is worthwhile and that this provides valid findings with regard to children’s stress regulation. It can be noted that LVET may serve as a good parameter to distinguish children of sensitive vs. insensitive mothers. Building on this, Köhler-Dauner et al. (2019) found that LVET shortened significantly with increasing disruptive behavior of the mother. We could speculate that the mother’s lower interaction quality may be less effective in engaging with the child from E1 on, which is reflected by the consistently low level of children’s LVET. Consequently, less sensitive mothers may not be able to address their children’s emotional and behavioral signals appropriately and fail to capture and maintain their children’s attention during interactions throughout the paradigm.

### Limitations

The limitation of our study lies first in its limited generalizability: characteristics such as the comparatively high level of maternal education and geographic location indicate that our sample cannot be deemed representative. Therefore, comparisons between our results and evidence presented by other studies can only be made with caution.

In addition, there were significantly more sensitive than insensitive mothers in the sample, which makes it difficult to draw conclusions about the children of insensitive mothers. Overall, the sample was relatively small, so it may not have been possible to detect any effects that might have existed. Therefore, a larger and more balanced sample should be sought in the future.

Also, the use of the EA Scales may have led to inaccurate estimates of interaction quality, as this is subjective to the raters’ judgments. Therefore, an additional measure could be applied in further studies to make more accurate assessments of interactions. For example, self- and peer-assessment questionnaires or, for slightly older children, an age-appropriate interview of these would be possible.

Furthermore, only four possible parameters of the ECG signal were considered in the context of this study. However, much more information exists that can be used in the context of an ECG study of ANS activity. Thus, this study did not examine the whole picture of ANS activation in relation to maternal subtlety, and more research is needed.

### Implications and Future Research

Based on the results discussed above, it can be concluded that RSA and LVET are appropriate physiological measures to distinguish the stress responses of children of sensitive vs. non-sensitive children. The HR, on the other hand, was not suitable for this purpose, at least in this sample. Therefore, other physiological parameters may exist to capture PNS and SNS activation.

Moreover, since no main effects of within comparisons of ANCOVAs emerged in this study, it should be considered whether the chosen paradigm is capable of inducing and releasing stress. Instead, additional tasks or paradigms could be developed and compared in further research. The use of other methods of measuring maternal sensitivity, such as questionnaires and interviews, should also be considered in future studies. Since the data were collected as part of a longitudinal study, further studies should also aim for a time series or comparison with previous surveys of similar parameters. Also, looking at other parameters for ANS activity is recommended in further research.

## Conclusion

Overall, it can be concluded that mother’s sensitivity can buffer the child’s stress levels at both, the SNS and PNS level. Regarding SNS activity, increased activation was shown in children of insensitive mothers in association with impaired PNS activity. As a consequence, child’s ANS balance may be impaired by low-quality parent interaction behavior which may result in long-term consequences on physical and mental health. Therefore, appropriate psychoeducational interventions for mothers of infants and young children should be developed. These should target especially mothers with regard to negative physical as well as psychological consequences of non-sensitive interactions for their child, where such difficulties have been observed in appropriate interactions, be it in the context of the childcare center, pediatrician, or other institutions.

## Data Availability Statement

The original contributions presented in the study are included in the article/supplementary material, further inquiries can be directed to the corresponding author.

## Ethics Statement

The study was approved by the Ethics Committee of Ulm University. The patients/participants provided their written informed consent to participate in this study.

## Author Contributions

FK-D, MG, and IM analyzed and interpreted the data regarding the effect of maternal interacting quality on child’s autonomic stress reactivity. All authors have read and approved the final manuscript.

## Funding

The study was funded by the Federal Ministry of Education and Research; Grant no. (01KR1304A); BMBF, 2013–2016, additional interim funding 2017.

## Conflict of Interest

The authors declare that the research was conducted in the absence of any commercial or financial relationships that could be construed as a potential conflict of interest.

## Publisher’s Note

All claims expressed in this article are solely those of the authors and do not necessarily represent those of their affiliated organizations, or those of the publisher, the editors and the reviewers. Any product that may be evaluated in this article, or claim that may be made by its manufacturer, is not guaranteed or endorsed by the publisher.

## Abbreviations

ANCOVA: Analysis of covariance
ANS: Autonomic nervous system
E1: Resting phase
E2: Structured play
E3: Free play
EA: Emotional Availability
ECG: Electrocardiogram
HR: Heart rate
ICG: Impedance cardiogram
LVET: Left ventricular ejection time
PEP: Pre-ejection period
PNS: Parasympathetic nervous system
RSA: Respiratory sinus arrhythmia
SNS: Sympathetic nervous system
